# Primary renal sinus tumor: Three case reports with a review of the literature

**DOI:** 10.3892/ol.2014.2729

**Published:** 2014-11-24

**Authors:** BO CHENG, QILIANG CAI, YUDONG WU, YAN ZHAO, QI GUO, GANG LI, XUENING ZHANG, AIMIN ZHANG, YUANJIE NIU

**Affiliations:** 1 Department of Urology, Shengli Oil Field Central Hospital, Dongying, Shandong 257034, P.R. China; 2 Department of Urology, The Second Hospital of Tianjin Medical University, Tianjin Institute of Urology, Tianjin 300211, P.R. China; 3 Department of Infection, The Second Hospital of Tianjin Medical University, Tianjin Institute of Infectious Disease, Tianjin 300211, P.R. China; 4 Department of Radiology, The Second Hospital of Tianjin Medical University, Tianjin 300211, P.R. China

**Keywords:** renal sinus, tumors, treatment

## Abstract

The aim of the present study was to investigate the clinical characteristics and management of primary renal sinus tumors. We retrospectively analyzed three cases of primary renal sinus tumors. The first patient was a 33-year-old man who presented with right flank pain for 6 months. Based on the imaging results, the patient was diagnosed with renal sinus tumor. The second case was a 34-year-old woman who presented with sudden lumbago in the right flank for 3 days. The imaging results confirmed the diagnosis of right renal angiomyolipoma. The third case was a 55-year-old woman with flank pain, which had persisted for 1 year. The imaging tests revealed lipoma of the left renal sinus. All three cases underwent surgical procedures. The first case was diagnosed with benign angioleiomyoma following pathological analysis. During surgery, the tumor was ablated and the kidney was spared. The second case was scheduled for tumor enucleating, but a nephrectomy was performed due to serious hemorrhaging and a damaged renal pelvis. Pathological analysis identified angiomyolipoma. The third case was scheduled for lipoma enucleating; however, nephrectomy was performed as the tumor encapsulated the renal vascular pedicle. Pathological analysis revealed lipoma. In the three cases, no relapse over 3 years, 10 months and 4 years of follow-up, respectively, was observed. In addition, this review examined previous literature and concluded that the occurrence of tumors in the renal sinus is rare and the majority of such tumors are benign. Furthermore, cases are easily misdiagnosed as renal pelvic tumors. Computed tomography, magnetic resonance imaging and intravenous urography are the best imaging examination methods for differential diagnosis. In conclusion, surgery is the usual approach for the treatment of renal sinus tumors and radical nephrectomy should be performed for malignant tumors.

## Introduction

Tumors arising from the renal sinus are rare. To the best of our knowledge, only three cases of tumors arising from the renal sinus have been reported in the literature to date (1–3). In 1999, Amin *et al* (1) reported the first known case of a renal sinus tumor with pathologogy of a myelolipoma. Hemangiomas, liposarcomas and myelolipomas have been found originating from the renal sinus. This report presents three cases of renal sinus tumors: Angioleiomyoma, angiomyolipoma and lipoma, respectively. Currently they are difficult to diagnose using imaging alone when they originate in this location and biopsies may not yield a definitive answer. Management options include both conservative and surgical approaches depending upon the certainty of the diagnosis, the progression of the patient’s symptoms and evidence of growth. Writted informed consent was obtained from all patients.

## Case reports

### Case 1

**Figure 1:**
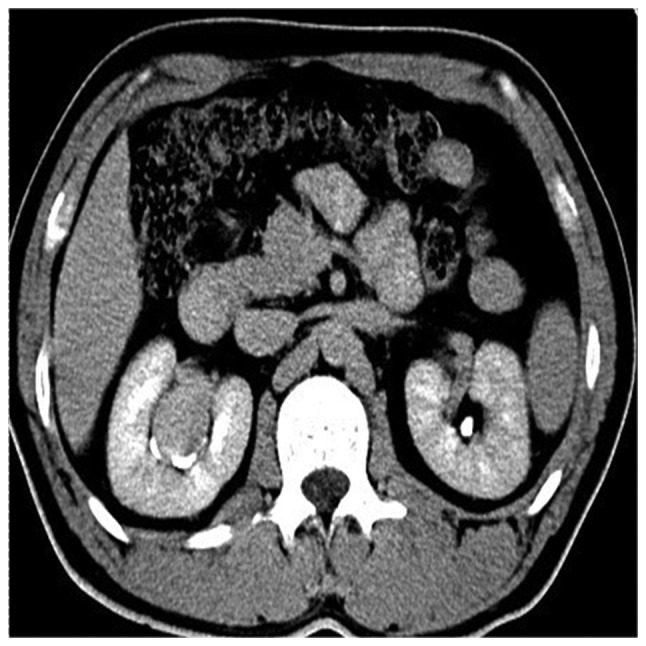
Case 1. Computerized tomography scan of the abdomen revealed a mild-enhancing, homogenous, smooth and evident mass, measuring ~3.5×3.7×3.0 cm.

A 33-year-old man presented to the Shengli Oilfield Central Hospital (Dongying, China) with right flank pain for 6 months. Ultrasonographic observation demonstrated a mass measuring ~3.5 cm in diameter in the right renal sinus. A computerized tomography (CT) scan of the abdomen revealed a non-enhancing, homogeneous, smooth and obvious mass, which measured ~3.5×3.7×3.0 cm in size (Fig. 1). Based on the results of the CT scan, the patient was diagnosed with a pelvic tumor. Intravenous urography (IVU) showed that the right renal pelvis and renal calyx were compressed, but the filling defect was not observed in the renal pelvis. All these changes suggested that the tumor mass may have arisen from the right renal pelvis. Surgical exploration of the renal sinus tumor was performed. An entity mass that was well-circumscribed and ~3.5 cm in diameter was situated in the renal sinus, and was adherent to the renal capsule and pelvis. Frozen tissue section examination revealed that the tumor was benign and originated from the mesenchymal tissue. Radical resection of the neoplasm was performed and normal renal tissues remained. Postoperative pathology results revealed that the tumor was well-circumscribed with well-differentiated smooth muscle cells and thick-walled vessels. Immunohistochemistry analysis showed positive staining for vimentin, smooth muscle actin, actin, CD34 and negative staining for Des, S-100 and neuron-specific enolase. The patient was diagnosed with angioleiomyoma due to the pathology results. The patient has been followed up for 3 years and has demonstrated no recurrences or any other problems associated with this lesion.

### Case 2

**Figure 2:**
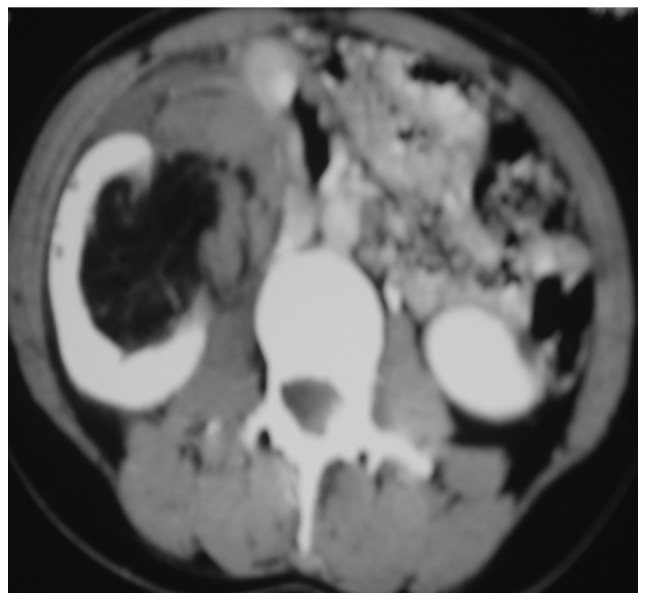
Case 2. Computed tomography (CT) scan of the kidney, demonstrating an oval and low-attenuation mass situated in the right renal sinus. The mass measured ~8×6×8 cm and had a CT value of −70 HU. Contrast-enhanced CT scan showed an enhanced heterogeneous mass in the right kidney.

**Figure 3:**
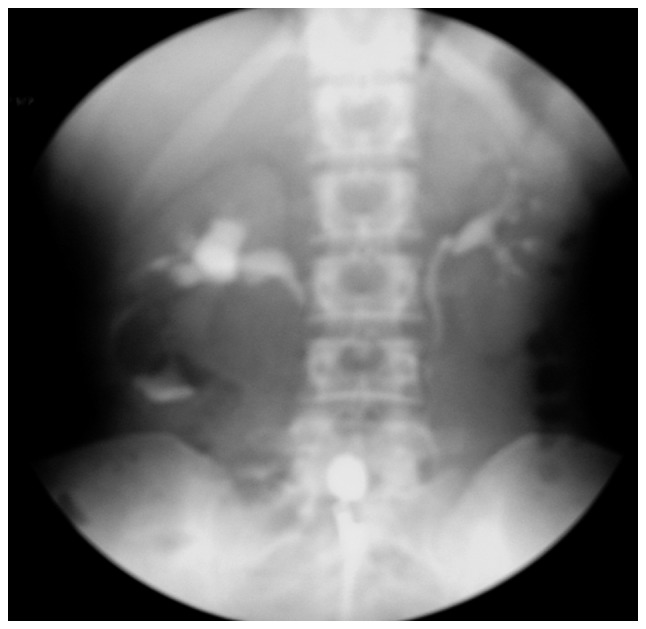
Case 2. Intravenous urography showed the lower collective system was compressed. It was without filling defects in the right renal pelvis.

A 34-year-old woman was referred to Shengli Oilfield Central Hospital due to sudden lumbago in the right flank with gross hematuria for 3 days on July 25, 2007. Right upper flank tenderness and percussion pain in the right kidney were observed during physical examination. The ultrasonography results of the right kidney demonstrated a hyperechoic mass in the right renal sinus, which measured ~8.4×5.5×8.5 cm in size. An oval and low-attenuation mass with a CT value of −70 HU in the right kidney sinus was identified in the CT scans, which was ~8×6×8 cm in size. An enhancing heterogeneous mass situated in the right kidney (Fig. 2), right renal capsular thickening, low density and subcapsular mass were observed in the contrast-enhanced CT scans. IVU results showed the lower collective system was compressed without filling defects in the right renal pelvis (Fig. 3). The left renal function was good, although accompanied with delayed right kidney enhancement. The patient was diagnosed with renal angiomyolipoma of the right kidney and right renal tumor resection was performed. The tumor adherent to surrounding tissues was ~8 cm in diameter and was situated in the right renal sinus. The patient underwent right kidney excision due to uncontrolled diffuse hemorrhage, and damage to the right renal pelvis could not be repaired. Postoperative pathology reports confirmed the diagnosis of angiomyolipoma of the right kidney. The patient exhibited a good physical condition and left the hospital after 7 days. The patient has been followed up for 10 months and has shown no recurrences or metastases.

### Case 3

**Figure 4:**
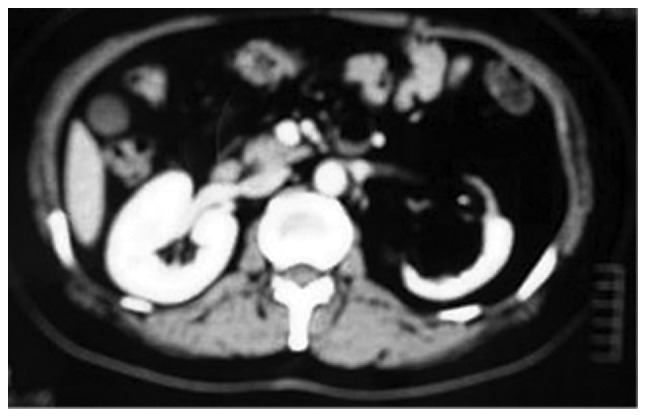
Case 3. Contrast-enhanced computed tomography (CT) scan of the abdomen showed a non-enhancing, homogenous and obvious mass measuring 8×5×5 cm in the left renal sinus with a CT value ranging from −50 HU to −35 HU.

**Figure 5:**
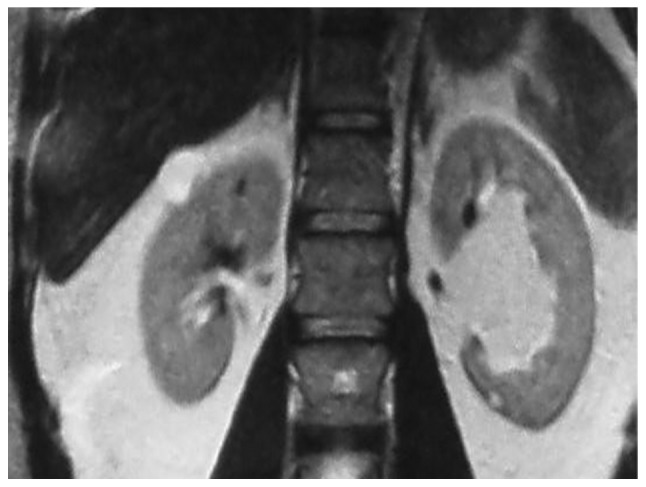
Case 3. Coronal magnetic resonance imaging examination of the kidney revealed an 8×5×5 cm lesion situated in the left renal sinus. Results showed a high signal on T1 weighted image (WI) and T2WI, but a low signal in fat suppression imaging.

A 55-year-old woman presented to the Second Hospital of Tianjin Medical University (Tianjin, China) with lumbago in the left flank, which had persisted for 1 year. Upon physical examination, tenderness in the left flank was observed. B-mode ultrasonography showed hyperechoic lesions in the left renal hilum. Contrast-enhanced CT scan of the abdomen showed a non-enhancing, homogenous and obvious mass in the left renal sinus, measuring 8×5×5 cm in size with a CT value ranging from −50 HU to −35 HU (Fig. 4). IVU results showed the collective system of the left kidney was compressed. Magnetic resonance imaging (MRI) showed a high signal on T1 weighted image (T1WI) and T2WI, but a low signal in fat suppression imaging (Fig. 5). The patient was diagnosed with lipoma of the left renal sinus. A left radical nephrectomy was performed. The tumors all formed a solid, large, smooth and evident mass. The cut surface of the tumor was yellow. Postoperative pathological reports confirmed the diagnosis of lipoma of the left renal sinus. The patient has been followed up for 4 years and has shown no recurrences.

## Discussion

The renal sinus is a cavity within the kidney containing the pelvis and calyces, adipose tissue, kidney vessels, nerves and lymphatic tissues, and is a continuation of the renal hilum. The types of tumor tissues in the renal sinus are extensive, including fat, lymphatic, nerve and vascular tissues. Lipomas and liposarcomas originate from fat tissue, Castleman’s disease and lymphoma originate from lymphatic tissue, ganglion cell tumors originate from nerve tissue, and angioleiomyoma and hemangiopericytoma originate from vascular tissue (4,5).

Tumors arising from the renal sinus are rare and two-thirds of such tumors are benign (6). The most common type of tumor arising from the renal sinus is lipoma (7,8). Previous studies have identified that the exclusive differential diagnosis of lesions or those involving the renal hilum includes hemangioma, angiomyolipomas, leiomyoma, myeloid lipoma, Castleman’s disease and Masson’s tumors (7,9–11). Malignant tumors, such as liposarcoma, non-Hodgkin’s lymphoma and malignant paragangliomas have occasionally been reported (12–14).

By virtue of the association of adipose tissue, imaging techniques, such as CT and MRI, can exaggerate internal enhancing or low attenuation and therefore may strongly suggest the diagnosis of renal sinus tumors. Simultaneously, diagnosis of renal sinus tumors mainly depends on the imaging results. Ultrasonography reveals occupying lesions, or blood vessels. CT or MRI scans are used to evaluate the developing stage and invasive range of the neoplasm. Multidetector CT offers a faster analysis, due to a thinner layer of scanning and a higher spatial resolution (15). Furthermore, three-dimensional reconstructive CT scans can more accurately determine lesions in the scope of complex renal sinus lesions compared with other imaging methods. The CT features of primary renal sinus tumors are as follows: i) commonly located within the renal sinus, and the renal parenchyma and pelvis may be encroached by a malignant tumor; ii) an obvious boundary between tumors and the renal collective system, with frequently compressed pelvis and calyces, and a lighter renal seeper; iii) enhanced CT scanning demonstrates compressed vessels situated in the renal hilum and iv) the excretion period of the enhancing CT scan reveals compressed renal pelvis and calyceal without filling defects (16–18). MRI imaging has more advantages than CT in revealing the invasion of the renal vein and inferior vena cava. This technique may be used in patients with kidney failure and in those with allergies using contrast agents. IVU may also be used to assess whether the tumor has affected the kidney collecting system and kidney function. Primary renal sinus neoplasms are often accompanied by a compressed collecting system (19–21). The absence of filling defects in the pelvis and calyces can be used to distinguish a primary renal pelvis tumor. It has been suggested that malignant tumors accompany the renal pelvis infringement when the kidney collecting system is irregular (22,23). One patient in the present study was diagnosed with a renal pelvis tumor, which was thought to have originated from the renal sinus and without renal pelvis filling defect. The combined application of IVU, CT and MRI scans may improve the diagnosis rate (15). It is difficult to obtain a qualitative diagnosis of renal sinus tumors, while angioleiomyomas and angiomyolipomas are markedly enhanced (11). In the present study, two patients were diagnosed with renal sinus angiomyolipomas and one was diagnosed with lipoma based on the appearance of adipose tissues and the CT values (approximately −70 and −50 HU, respectively).

The clinical manifestations of renal sinus tumors are nonspecific; a number of patients present with lumbago and a few patients present with hematuria and urinary tract infections (24–26). Symptoms present when the diameter of the tumor is >4 cm. Hematuria was observed to be caused when the tumor was larger and accompanied with renal pelvis involvement. To the best of our knowledge, no symptoms for benign tumors have been observed when the diameter of the tumor is <4 cm; therefore, surgery is unnecessary before the appearance of renal seeper. Tumors with apparent symptoms and a diameter >4 cm should be resected. In the present three cases, we preserved the kidney as far as possible during surgery of the three reported cases. Previously reported neoplasms required kidney resection when the diameter was >7 cm, particularly benign tumors that occupied the entire kidney sinus (27). In the present study, two cases demonstrated the same condition; the tumor diameter was >7 cm and occupied the entire kidney sinus, involving the renal pelvis or with serious adhesion. Therefore, nephrectomy was performed. Renal sinus tumors would remain undiagnosed unless the possibility of hemangioma via percutaneous or intraoperative biopsy is excluded (2). In the current study, one patient was diagnosed with a benign tumor based on the intraoperative biopsy results and underwent excision of the tumor, whilst simultaneously, preserving the kidney. Therefore, surgical exploration is advocated before excluding the possibility of a renal sinus tumor, in order to avoid unnecessary nephrectomy. Renal sinus malignant tumors, particularly those with renal parenchyma infringement, should undergo radical nephrectomy where the kidneys are removed simultaneously with the with tumor emboli (3).

In conclusion, primary tumors in the renal sinus are rare and the majority are benign, but are often misdiagnosed as renal pelvic carcinoma. The prognosis of renal sinus tumors is good. CT, MRI and IVU analysis have great value in orientation and qualitative diagnosis of renal sinus tumors. The treatment approaches, however, may be established by correlating the clinical symptoms, tumor size and nature. Preserving the kidney during surgery as far as possible is key for the treatment of patients with renal sinus tumors.
